# Living With Early-Onset Dementia in China: Through a Person-Centered Care Lens

**DOI:** 10.1093/geroni/igab046.252

**Published:** 2021-12-17

**Authors:** Jing Wang, Ding Ding, Qianhua Zhao

**Affiliations:** 1 Fudan University, Shanghai, Shanghai, China (People's Republic); 2 Fudan University Affiliated Huashan Hospital, Shanghai, Shanghai, China (People's Republic); 3 Fudan University Affiliated Huashan Hospital, shanghai, Shanghai, China (People's Republic)

## Abstract

We conducted semi-structured interviews with 35 dyads of persons with early-onset dementia (EOD) and their primary informal care partners to explore their dyadic experiences of living EOD in Shanghai, China. Many of them are in their 50s and still need to make familial, financial, and social commitments. They experienced significant disruptions of their "normal" family life and family dynamics, social stigma, and felt marginalized when there was very limited age-appropriate support for them. During COVID-19 pandemic, many persons with EDO and their care partners had decreased social networking opportunities, physical exercises and experienced an increased level of social isolation. The pandemic further complicated their family dynamics, relationships, and communications. Care partners used their strengths to adaptively deal with multiple challenges, cope with the stress, social isolation, and normalize their family life by facilitating collaborative work with persons with EOD.

